# Computer-assisted Taylor spatial frame for correcting malalignment after unilateral external fixation of tibial fractures

**DOI:** 10.1097/MD.0000000000051508

**Published:** 2026-11-06

**Authors:** Zhiming Zhao, Chengkuo Cai, Guoqi Ji, Weiguo Xu

**Affiliations:** a Department of Traumatic Orthopaedics, Tianjin Hospital, Tianjin University, Tianjin, China.

**Keywords:** computer-assisted reduction, malalignment, Taylor spatial frame, tibial fracture, unilateral external fixation

## Abstract

This study aimed to evaluate the clinical efficacy of computer-assisted Taylor spatial frame (TSF) for correcting malalignment after unilateral external fixation (UEF) of tibial fractures. This retrospective cohort study included 18 patients with tibial misalignment after UEF between February 2019 and February 2024. Using the TSF technique, X-ray parameters (deformity, frame, and mounting) were entered into software to generate a six-axis correction prescription. Reduction time, number of X-ray exposures, total external fixation duration, clinical fracture healing time, complications, and functional recovery were recorded. The Wilcoxon signed-rank test was used to compare preoperative and final visual analogue scale (VAS) scores because the paired differences were not normally distributed; data are presented as mean (range) or median (interquartile range [IQR]). All 18 patients were followed up for a mean of 14.9 months (range: 12–18 months). Satisfactory reduction was achieved in all cases after TSF adjustment. Mean reduction time was 19.0 minutes (range, 15–24 minutes), and patients received a mean of 1.4 X-ray exposures (range, 1–3). Clinical fracture healing occurred after a mean of 24.0 weeks (range, 21–29 weeks), and total external fixation lasted a mean of 25.2 weeks (range, 22–30 weeks). Johner-Wruhs ratings at final follow-up were excellent in 15 patients and good in 3 (100% excellent/good). Median VAS scores improved from 7 (IQR, 6–8) before surgery to 1 (IQR, 0–2) at final follow-up (Wilcoxon signed-rank test, *Z *= −3.77, *P* < .001). Superficial pin tract infections developed in 3 patients (16.7%; 3/18) and were controlled with oral antibiotics and dressing changes. No patient had deep infection, pin loosening, nonunion, or bone exposure. In this small, single-arm retrospective cohort, all 18 patients achieved satisfactory reduction with computer-assisted TSF, and adjustment times were short. No patient underwent secondary open reduction or fixation. These preliminary findings suggest that TSF may serve as a feasible minimally invasive option for correcting early malalignment following UEF of tibial fractures; however, multicenter controlled studies with longer follow-up are needed to confirm these results.

## 1. Introduction

Tibial fractures are among the most common lower limb fractures in clinical practice and often result from high-energy trauma. Their anatomical location and limited soft tissue coverage make them susceptible to complications, including open injuries, vascular and nerve damage, and severe soft tissue defects, which complicate treatment.^[1,2]^ UEF is widely used for the initial fixation of open tibial fractures, but its eccentric design provides limited resistance to rotation and angular deformation. This design limitation contributes to postoperative malalignment, which can impair limb function and complicate subsequent treatment.^[3–5]^ Previous studies have reported malalignment rates of approximately 10% and 20% after UEF for tibial fractures, with angular deformities being the most common type.^[3]^

Conventional revision strategies have limitations. Manual reduction is often insufficient for precise correction of multidimensional deformities. Conversion to internal fixation or a circular external fixator requires a second operation, which may increase the risks of infection and nonunion.^[6–8]^ The Taylor spatial frame (TSF) is a computer-assisted, six-axis circular external fixator that enables precise correction of complex deformities through parametric adjustments.^[5,9,10]^ Its six telescopic struts provide multiplanar stability and allow simultaneous correction of angulation, rotation, translation, and length. The computer-generated prescription permits gradual, controlled adjustment at the bedside without anesthesia or repeated surgery. These features may be particularly useful for patients with compromised soft tissue conditions during the early postoperative period.^[3,9]^ However, evidence on the corrective value of the TSF in the early management of malalignment following UEF remains unclear.

To date, published studies on TSF have mainly focused on its use as primary fixation for acute fractures, as well as on the correction of established bony deformities and malunions.^[5,10]^ However, whether TSF can serve as an early, minimally invasive adjustment tool for fresh malalignment following UEF has rarely been reported. This study, therefore, evaluated the feasibility of using TSF as an early, minimally invasive adjustment tool for malalignment following UEF.

## 2. Methods

### 2.1. Study design, setting, and population

This was a retrospective cohort study conducted at Tianjin Hospital, Tianjin University. The study period was February 2019 to February 2024. We retrospectively screened the hospital’s electronic clinical database for all patients with tibial fractures who underwent UEF during this period. The medical records were reviewed according to the inclusion and exclusion criteria described below. A total of 33 patients were initially identified. Of these, 15 patients were excluded for the following reasons: age outside 21 to 60 years (n = 2); deformity did not meet the threshold (n = 4); interval from UEF to TSF > 6 weeks (n = 3); fewer than 2 half-pins on the unilateral side (n = 2); severe cardiovascular, cerebrovascular, or respiratory disease (n = 2); incomplete follow-up data (n = 1); and follow-up duration < 12 months (n = 1). Finally, 18 consecutive eligible patients were included in the analysis. All included patients completed at least 12 months of follow-up.

Among the 18 included patients, 10 were male and 8 were female, with a mean age of 36.7 years (range: 21–60 years). Fractures were located on the left side in 7 cases and on the right side in 11 cases. There were 11 cases of closed tibial fractures (AO/OTA classification: 3 cases of type 42A1, 2 cases of type 42A2, 3 cases of type 42A3, one case of type 42B2, and 2 cases of type 42B3). In one of these cases, compartment syndrome of the lower leg developed, requiring fasciotomy; the wound healed after secondary closure following resolution of swelling. Seven open tibial fractures were classified according to the Gustilo-Anderson system as type I (one case), type II (two cases), type IIIA (two cases), type IIIB (one case), and type IIIC (one case). Among these, four achieved primary wound healing after debridement, exploration, and suturing; 2 with skin necrosis healed after skin grafting; and one with bone exposure healed after local fasciocutaneous flap transfer. The main types of deformity included anterior angulation in 3 cases, posterior angulation in 2 cases, varus deformity in 2 cases, valgus deformity in 3 cases, internal rotation deformity in one case, and combined deformities in 7 cases. The interval from UEF to TSF-assisted fracture reduction ranged from 3 to 42 days, with a mean interval of 19.7 days (Table 1).

**Table 1:** Demographics of patients.

Case	Age (years)	Gender	Injured side	Types of deformities	Types of fractures		Interval(days)
					Closed Fracture (AO/OTA)	Open Fracture (Gustilo-Anderson)	
1	56	Male	Left	Anterior angulation	42A2	–	26
2	25	Male	Right	Complex	42B3	–	18
3	35	Male	Left	Complex	–	Type II	17
4	24	Female	Left	Posterior angulation	42A1	–	11
5	36	Male	Right	Complex	42A3	–	3
6	27	Male	Left	Valgus	–	Type I	34
7	31	Male	Right	Anterior angulation	42A1	–	8
8	45	Female	Right	Valgus	42B3	–	36
9	28	Male	Left	Varus	–	Type IIIA	8
10	31	Male	Right	Posterior angulation	42A1	–	24
11	47	Female	Right	Complex	42B2	–	31
12	31	Male	Right	Internal rotation	–	Type II	13
13	42	Female	Right	Anterior angulation	42A2	–	22
14	52	Female	Right	Varus	–	Type IIIB	42
15	60	Female	Left	Complex	42A3	–	8
16	42	Female	Right	Complex	–	Type IIIA	29
17	21	Female	Left	Valgus	–	Type IIIC	19
18	28	Male	Right	Complex	42A3	–	5

AO/OTA = Arbeitsgemeinschaft für Osteosynthesefragen/ Orthopaedic Trauma Association, UEF = unilateral external fixation, TSF = Taylor spatial frame.

Inclusion Criteria: Age between 21 and 60 years; tibial fractures (both open and closed) with malalignment following UEF; angular deformity: > 5° varus/valgus in the coronal plane or > 5° procurvatum/recurvatum in the sagittal plane; rotational deformity: >10° internal/external rotation; displacement: >3 mm lateral translation; gap/shortening: >5 mm.^[11]^ Time from UEF to the application of computer-assisted TSF ≤ 6 weeks. Exclusion criteria were as follows: insufficient number of fixation pins used at the fracture site during initial fixation (fewer than 2 half-pins on the unilateral side); severe cardiovascular, cerebrovascular, or respiratory diseases that could not tolerate surgery or increased perioperative risks; incomplete follow-up data; or follow-up duration of <12 months. The study was approved by the Medical Ethics Committee of Tianjin Hospital (Approval No. 2026108) and was conducted in accordance with the Declaration of Helsinki. Because this was a retrospective study, all patients were exempted from signing informed consent.

### 2.2. Surgical technique

The TSF was installed at the bedside. The patient was placed in the supine position on a sterile bed in the dressing room, and the entire procedure was performed without anesthesia. Two full rings or 2/3 rings were positioned at both ends of the fracture. Six appropriately sized rapid-fit Taylor rods were installed between the 2 rings. Half-pins were connected to the rings using connecting plates and half-pin clamps. Tibial anteroposterior (AP) and lateral radiographs were obtained to assess the type of tibial fracture malalignment (including angulation, rotation, and displacement) and to obtain malalignment, frame, and mounting parameters. These parameters were input into the CareFix-TSF computer software (Shanghai Carefix Medical Instrument Co., Ltd., Shanghai, China), which generated a six-axis correction electronic prescription. The bolts of the UEF were loosened, and the fracture was reduced by adjusting the lengths of the Taylor rods. The TSF was adjusted once or multiple times. X-ray images were reviewed to confirm the reduction effect. After satisfactory reduction had been achieved, the UEF bolts were tightened before TSF removal.

### 2.3. Postoperative management

After surgery, the external fixator pin tracts were disinfected daily with 75% ethanol and kept dry, and the dressings were changed regularly. The pin sites were monitored for infection, and superficial pin tract infections were treated with oral antibiotics and dressing changes. Patients with concomitant soft tissue injuries also received regular dressing changes. Skin grafting or flap reconstruction was performed as a second-stage procedure once wound conditions were suitable. Rehabilitation began immediately postoperatively and focused on knee and ankle mobilization. From week 3 onward, partial weight bearing with bilateral crutches was permitted. Weight bearing then increased gradually according to pain tolerance, and full weight bearing with bilateral crutches was permitted by week 5. Crutches were discontinued between weeks 7 and 8 after radiographs indicated fracture healing.

Follow-up occurred monthly and comprised a clinical examination and standard AP and lateral radiographs. Once clinical fracture healing had been confirmed, the frame was loosened in the outpatient clinic, and patients were instructed to walk with the loosened frame for approximately one week. If the fracture site remained pain-free while the fixator was no longer load-bearing, the frame was removed under sterile conditions in the operating room with the patient under anesthesia.

### 2.4. Evaluation indicators

To ensure consistency, the following definition of satisfactory reduction was used throughout the manuscript: angulation ≤ 5° in each of the coronal and sagittal planes, internal/external rotation ≤ 10°, lateral displacement ≤ 3 mm, separation or shortening ≤ 5 mm, and restoration of the tibial mechanical axis.^[12]^ Clinical fracture healing required bridging callus across at least 3 of the four cortices on standard AP and lateral radiographs, together with absence of fracture-site pain. Finally, the number of X-ray exposures was defined as the number of radiographic examinations performed to assess reduction. Each examination included an AP and a lateral view obtained together and was therefore counted as one exposure.

We evaluated reduction time, the number of X-ray exposures, total external fixation duration, and time to clinical fracture healing. Reduction time was defined as the interval from the start of TSF installation until TSF removal was complete. TSF removal was performed only after satisfactory reduction had been confirmed radiographically and the UEF bolts had been tightened. The procedure began with TSF installation, after which the fracture was reduced by adjusting the Taylor rods. X-rays confirmed the reduction, after which the UEF bolts were tightened and the TSF was removed. At the final follow-up, functional results were graded with the Johner-Wruhs^[13]^ criteria for tibial fractures. An excellent result meant no pain, joint range of motion > 75% of the unaffected side, normal gait and alignment, and return to the original work. A good result meant only occasional activity-related pain, range of motion > 50%, a mild gait problem without orthotic support, and alignment deviation < 5°. A fair result meant pain requiring medication, range of motion < 50%, walking with orthotic support, and malalignment of 5°–10°. A poor result meant persistent pain, joint stiffness, malalignment > 10°, or a need for secondary surgery.

Total external fixation duration refers to the total time (in weeks) from the initial application of the UEF to its final removal, regardless of whether complete healing was achieved at the time of the removal. Time to clinical fracture healing denotes the duration (in weeks) from the initial application of the UEF until the fracture meets the criteria for clinical fracture healing.

The VAS is a common tool for pain assessment. It is a 10-cm sliding ruler with 10 gradations, and the 2 ends stand for scores of 0 and 10. A score of 0 indicates no pain, scores of 1 to 3 denote mild pain, scores of 4 to 6 indicate moderate pain, and scores of 7 to 10 represent severe pain.

Radiographic assessment: All radiographic measurements were performed independently by 2 orthopedic surgeons (ZZ and CC) using a picture archiving and communication system (PACS). Measurements were made on standard AP and lateral tibial radiographs. Angulation was measured as the angle between the anatomical axes of the proximal and distal tibial segments on AP radiographs (coronal plane, varus/valgus) and lateral radiographs (sagittal plane, procurvatum/recurvatum). Rotation was assessed by clinical examination and recorded as the degree of internal/external rotation relative to the contralateral limb. Displacement was measured as the maximum lateral translation of the fracture fragments on AP and lateral radiographs. Shortening was measured as the difference in tibial length between the injured and contralateral limbs on full-length radiographs, or as the amount of overlap at the fracture site when contralateral imaging was unavailable.

### 2.5. Statistical analysis

Statistical analyses were performed using SPSS version 26.0 (IBM Corp., USA). All VAS scores were paired observations because preoperative and final follow-up assessments were obtained from the same patients. The paired differences were assessed for normality using the Shapiro–Wilk test. Because the paired differences were not normally distributed (Shapiro–Wilk test, *P* = .001), the Wilcoxon signed-rank test was used to compare preoperative and final follow-up VAS scores. Continuous variables are presented as mean (range) or median (interquartile range [IQR]), as appropriate. Categorical variables are presented as frequencies, with percentages where appropriate. A two-sided *P* value < .05 was considered statistically significant.

## 3. Results

This retrospective cohort comprised 18 patients who completed follow-up, with a mean follow-up duration of 14.9 months (range, 12–18 months). At the final follow-up, radiographs showed bony union in all patients. After TSF adjustment, satisfactory reduction of the fracture was achieved in all patients, with an average reduction time of 19.0 minutes (range: 15–24 minutes), an average number of X-ray exposures of 1.4 (range: 1–3), an average clinical fracture healing time of 24.0 weeks (range: 21–29 weeks), and an average total external fixation duration of 25.2 weeks (range: 22–30 weeks). According to the Johner-Wruhs scoring system,^[13]^ the outcomes were rated as excellent in 15 cases and good in 3 cases, yielding an excellent and good rate of 100%. No complications, such as deep pin tract infections, loosening of fixation pins, nonunion, or bone exposure, were observed (Table 2). The median VAS score decreased from 7 (IQR, 6–8) preoperatively to 1 (IQR, 0–2) at the final follow-up (Wilcoxon signed-rank test, *Z* = −3.77, *P* < .001; Table 3). Superficial pin tract infection occurred in 3 of 18 patients (16.7%) and was controlled with oral antibiotics and dressings. A typical case is shown in Figure 1.

**Table 2:** Clinical data and outcomes.

Case	Reduction time (min)	Number of X-ray exposures	Clinical fracture healing time (week)	Total external fixation duration (week)	Follow-up time (month)	Complications	Johner-Wruhs criteria	VAS	
								Preoperative	Final follow-up
1	20	1	24	25	16	–	Excellent	5	0
2	18	1	21	22	18	–	Excellent	8	2
3	17	2	22	23	16	–	Excellent	7	1
4	15	1	25	26	12	–	Excellent	7	1
5	17	3	24	25	15	–	Excellent	5	0
6	19	1	26	28	17	–	Excellent	8	3
7	20	1	21	22	14	–	Excellent	8	0
8	22	2	24	27	15	Superficial pin tract infection	Good	7	2
9	18	1	25	26	13	–	Excellent	7	0
10	16	1	23	24	18	–	Excellent	7	1
11	20	2	25	26	16	Superficial pin tract infection	Good	8	0
12	21	1	25	26	12	–	Excellent	8	0
13	24	1	22	23	13	–	Excellent	8	1
14	21	3	29	30	17	–	Excellent	6	1
15	18	1	25	26	14	Superficial pin tract infection	Excellent	7	2
16	17	1	26	27	13	–	Excellent	6	1
17	23	1	23	24	14	–	Good	9	3
18	16	2	22	23	15	–	Excellent	6	1

VAS = Visual analogue scale.

**Table 3:** Comparison of preoperative VAS and final follow-up VAS.

Variable	n	Median (IQR)	*Z* value	*P* value
**Preoperative VAS**	18	7 (6–8)	−3.77	<.001
**Final follow-up VAS**	18	1 (0–2)		

Data are presented as median (interquartile range [IQR]). Preoperative and final follow-up VAS scores were compared using the Wilcoxon signed-rank test. VAS = visual analogue scale. A two-sided *P* value < .05 was considered statistically significant.

**Figure 1.**
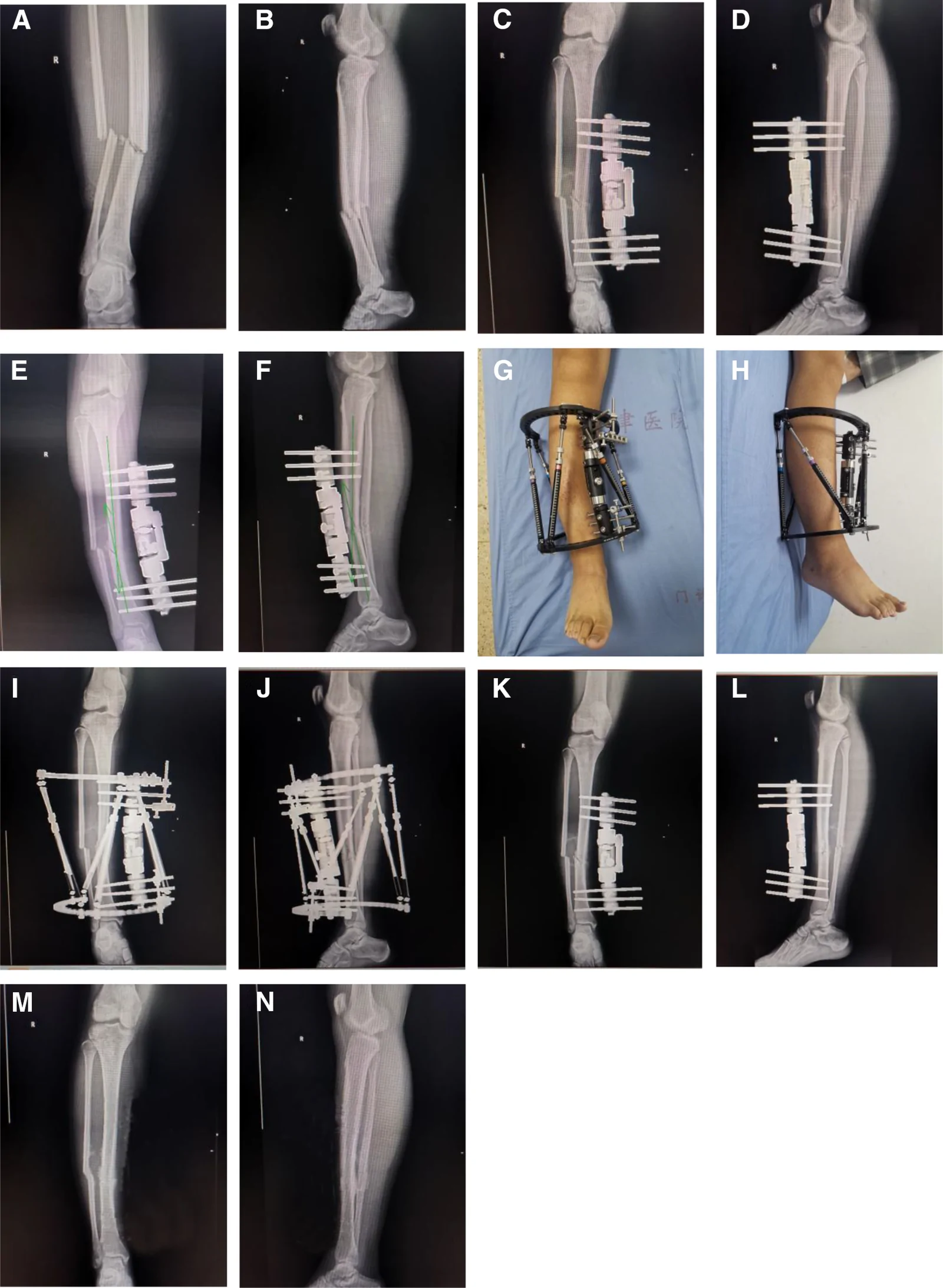
Images of treating a 28-year-old male with malalignment after unilateral external fixation for tibial fracture using computer-assisted TSF. (A and B) Preoperative radiographs of the injured tibial fracture. (C and D) Postoperative radiographs revealing satisfactory reduction of the tibial fracture. (E and F) Anterior angulation and varus deformity occurred after unilateral external fixation for tibial fracture. (G and H) TSF was installed on the affected limb before the tibial fracture was reduced. (I and J) Postoperative radiographs after TSF-assisted reduction of the tibial fracture, showing the satisfactory reduction achieved. (K and L) Postoperative radiographs revealing satisfactory reduction of the tibial fracture after TSF was removed. (M and N) AP and lateral radiographs show good fracture healing 1 week after removal of UEF.

## 4. Discussion

Tibial fractures are hard to treat, and this is especially true when they come from high-energy trauma, as seen in open fractures or when the soft tissue around them is badly damaged.^[1,14]^ For these fractures, UEF is a common initial fixation choice because it can be applied quickly, is minimally invasive, and provides early fracture stabilization.^[15]^ In our practice, however, its limitations often become apparent postoperatively. Because the fixator is mounted eccentrically, it provides limited resistance to rotation and angular deformation. As a result, angular, rotational, or displacement deformities may be observed during follow-up.^[4,16,17]^ Contributing factors include insufficient fixation strength, early weight bearing before callus formation, and inadequate initial reduction, either alone or in combination. When malalignment occurs, manual adjustment is commonly performed. But this seldom fixes a complex deformity in several planes at the same time. Conversely, transitioning to internal fixation (e.g., plates, intramedullary nails) or switching to circular external fixators necessitates waiting for improved soft tissue conditions, which carries drawbacks, including delayed treatment timing, increased trauma from secondary surgery, disruption of the fracture site’s blood supply, and potential infection risks.^[3,7,8]^ Therefore, there is a significant clinical need for a method capable of achieving precise and efficient correction of malalignment under minimally invasive conditions while avoiding additional major surgical interventions.

This study reports the use of computer-assisted TSF as a minimally invasive bridging strategy in this clinical setting. The observed outcomes suggest that this approach may mitigate the limitations described above. After TSF adjustments, all 18 patients got satisfactory reduction. Each adjustment took an average of only 19.0 minutes, and an average of only 1.4 X-ray exposures confirmed the reduction. A possible reason is the TSF system itself: it has a six-axis spatial structure, and a computer does the calculations.^[18–20]-^ In contrast to the trial-and-error adjustments often required for traditional circular fixators or UEFs, the TSF allows precise measurement and direct entry of malalignment parameters (displacement, angulation, and rotation), frame parameters (ring sizes and rod lengths), and mounting parameters (reference points). Dedicated software then generates an individualized electronic prescription. Complex deformity correction is thereby divided into six linear adjustments, each corresponding to a measurable change in the length of a single telescopic rod. These adjustments allow reduction to proceed in a controlled, stepwise manner under closed, minimally invasive conditions. The short reduction times and low X-ray exposure count in this cohort suggest a low procedural burden, although controlled comparisons with alternative strategies are still required.

Regarding treatment outcomes, the mean total external fixation duration for this cohort was 25.2 weeks, and all fractures had healed at the final follow-up. According to the Johner-Wruhs scoring system, the excellent and good rate was 100% (15 cases rated as excellent, 3 as good). Pain symptoms improved during follow-up, with the median VAS score decreasing from 7 (IQR, 6–8) preoperatively to 1 (IQR, 0–2) at the final follow-up (Wilcoxon signed-rank test, *Z* = −3.77, *P* < .001). In this cohort, TSF-assisted reduction corrected the deformities, and none of the 18 patients underwent secondary open reduction or internal fixation during the study period. This observation may be relevant to the preservation of the fracture site’s biological environment,^[4,21]^ although this mechanism was not directly assessed in the present study. Three of the 18 patients in this study developed superficial pin tract infections, and all of them cleared up with oral antibiotics and local wound care – none needed admission or removal of the pin. Nothing worse was seen: no deep infection, no loosening of the fixation pins, no nonunion, and no bone exposure occurred during the follow-up period. In our experience, the pin sites that needed the most attention were those crossing the region of soft-tissue swelling, where daily dressing changes were started early as a precaution.

The results we obtained are very much in line with what earlier authors have described. TSF has been reported to work well in a fairly wide range of problems, from congenital deformities to post-traumatic malunions and nonunions, and even as the primary external fixation for complex fractures; our own cases, whether measured by frame time or by fracture healing, behaved much the way these published series would predict.^[22–25]^ These studies have reported favorable outcomes with the TSF for complex multiplanar skeletal and joint deformities. The present study applied the technique in a different context: minimally invasive correction of tibial fracture malalignment that developed early after UEF, at a mean interval of 19.7 days (range, 3–42 days). Unlike a mature deformity with bony healing, which typically requires an osteotomy, this malalignment was corrected during the early inflammatory and repair phase (< 6 weeks), when it remained less established and potentially reversible, whether iatrogenic or secondary. The TSF served as a minimally invasive adjustment tool to correct malalignment and restore alignment. This approach aimed to preserve the initial UEF treatment plan and avoid escalation to more complex procedures, such as conversion to a circular external fixator or open reduction and internal fixation.

The external fixator remained in place for 25.2 weeks, 1.2 weeks beyond the time to clinical fracture healing (24.0 weeks). This delay was planned because clinical healing provides evidence of initial stability, whereas the callus may not yet be fully mature. To reduce the risk of reduction loss or refracture, we added a functional assessment period. After clinical healing was confirmed, the fixator bolts were loosened, and patients were instructed to walk with full weight bearing while the fixator remained in place for approximately 1 week. Fracture stability, patient tolerance, and pain response were assessed under physiological loading. The fixator was removed only if no adverse findings were identified. This approach extended fixation by 1.2 weeks but allowed functional assessment before final removal.

Our study has some limitations. First, its retrospective, single-center, single-arm design with a small sample (n = 18) and no control group may introduce selection bias and limit the generalizability of the findings. Second, the available 14.9-month follow-up may be insufficient to assess long-term complications such as post-traumatic arthritis. Third, only patients with UEF-to-TSF conversion ≤ 6 weeks were included, leaving TSF’s later deformity correction effectiveness unassessed. Fourth, pain and analgesia during TSF adjustment weren’t systematically recorded. Despite a short reduction time (19.0 minutes), the lack of pain data is a limitation. Future research should assess and manage pain. Fifth, the application of TSF entails a learning curve, and the precision of parameter measurement may influence therapeutic outcomes. Rotational deformity was assessed clinically using the thigh-foot angle rather than axial computed tomography, which may have limited the accuracy of rotational measurement. Large multicenter randomized controlled trials with longer follow-up and standardized training protocols are needed to validate these findings.

## 5. Conclusion

All 18 patients in this small, single-arm retrospective cohort achieved satisfactory reduction with computer-assisted TSF. Adjustment times were short, and no patient underwent secondary open reduction or fixation. These preliminary findings suggest that TSF may be a feasible minimally invasive option for the early correction of malalignment after UEF for tibial fractures. However, multicenter controlled studies with longer follow-up are needed to confirm these findings.

## Author contributions

**Writing – original draft:** Zhiming Zhao.

**Writing – review & editing:** Zhiming Zhao.

**Data curation:** Chengkuo Cai.

**Formal analysis:** Guoqi Ji.

**Supervision:** Weiguo Xu.
